# Advanced neonatal medicine in China: Is newborn ward capacity associated with inpatient antibiotic usage?

**DOI:** 10.1371/journal.pone.0219630

**Published:** 2019-08-13

**Authors:** Yi Ge, Selma Chipenda Dansokho, Xiang-Peng Liao

**Affiliations:** 1 Department of Pediatrics, Gongli Hospital, The Second Military Medical University, Shanghai, China; 2 Office of Education and Continuing Professional Development, Faculty of Medicine, Laval University, Quebec City, Quebec, Canada; University of Campania, ITALY

## Abstract

Previous surveys of neonatal medicine in China have not collected comprehensive information on antibiotic use in newborns. The goal of the present study was to assess the trends in antibiotic use in inpatient newborns from advanced hospitals in mainland China and to evaluate the contributing factors. We extracted retrospective data on newborn clinical units from a database containing key clinical subspecialty area indicators from provincial or ministerial (Class A level III) hospitals over three consecutive years (2008–2010) and in 25 of 31 provincial districts of mainland China. Fifty-five newborn units were included in the study. The results showed that two thirds (65.7% ± 23.1%) of inpatient newborns were prescribed antibiotic products. Antibiotic use rates were significantly different by newborn ward bed capacity (*p* = 0.023; 60.6% for d capacity (ficant65.7% ± 23–100 beds group, and 77.1% for (ficant65.7% ± 23.1%) of inpatient newb significantly different by type of hospital, geographic area, admission to physician or nurse ratio, or physician or nurse academic degree. Factors contributing significantly to antibiotic use included ward bed capacity, physician to nurse ratio, average hospital stay, and pneumonia to preterm infant ratio. Our data suggested that the use of antibiotics among inpatient newborns in advanced hospitals in mainland China was prevalent and should be subject to rigorous monitoring, and highlighted the need to explore how newborn ward bed capacity potentially impacts antibiotic use.

## Introduction

Overuse of antibiotics is now a major health concern worldwide, primarily due to the spread of antibiotic resistance and its public health consequences but also because of its impact on individual health outcomes[1]. Exposure to antibiotics during the early stages of life can alter the fetal and neonatal microbiome and increase the risk of adverse effects, such as obesity and asthma, in the short to long-term[2–4]. A study in 127 Neonatal Intensive Care Units (NICUs) across California showed that antibiotic use was independent of proven infection, surgical volume, or mortality, and that 50% of intermediate level NICUs had the highest antibiotic use quartile, yet their reported infection rates were very low (zero) in most of these units [5].

More reports of antibiotic use from China are related to adults and children than newborns. Results from a meta-analysis showed that country-wide, 50.3% of adult outpatients were prescribed antibiotics, with higher rates of prescription in the less developed areas of western China[6]. A national survey in primary health care settings in 2014 found that 52.9% of outpatients were prescribed antibiotics (only 39.4% were prescribed correctly), and 77.5% of inpatients received antibiotic therapy (only 24.6% were prescribed correctly)[7]. Children in China receive inpatient care in one of three types of hospital settings: children’s hospitals (CH), general hospitals (GH), and maternal and child hospitals (MCH). A national survey on inpatient antibiotic use in 2001 found that pediatric departments had the highest percent of antibiotic use for inpatients (83.4% for newborns and 84.0% for children)[8]. A survey conducted in three large CHs in 2006 found that 94.7% of inpatients in pediatric intensive care units received at least one antibiotic agent during their stay[9]. And a report also observed decreases of antibiotic use and changes in choosing antibiotic treatment in children[10]. However, more research is needed to examine antibiotic use among the youngest segment of the population and to monitor change, at the national level, over time.

Research suggests that there are a number of underlying factors that influence antibiotic prescription including, financial incentives provided by pharmaceutical industry[11, 12], a poor awareness among physicians and patients of the consequences of antibiotic overuse, and insufficient management and supervision for rational drug prescribing[7, 13–15]. At policy level, the development and implementation of antibiotic use guidelines[10, 16], the restructuring of the health care system to favor community health centres[17], and China’s essential medicines scheme[17, 18], are thought to influence antibiotic prescribing behavior. However, more studies are needed to identify the main contributing factors and to develop appropriate policies and interventions to optimize the use of antibiotics.

In 2011, three ministries including National Health and Family Planning Commission of the People’s Republic of China (NHFPC) initiated a national initiative to develop key clinical subspecialties for the country[19]. We used data from the survey and developed a national database on health care service and research on advanced neonatal medicine in China in 2017[20, 21]. In this article, we use the survey data to examine the prevalence of antibiotic use over a span of three consecutive years (2008, 2009, and 2010), and explore potential contributing factors.

## Methods

### Data sources

We used retrospective cross-sectional data covering a period of three consecutive years (2008–2010). Data were extracted from proposals submitted to the NHFPC. All participating hospitals were required to provide detailed information, including indicators of institutional infrastructure, workforce, health-care service, education, and research[20, 21]. The information gathered was validated using supporting documentation and field checks. Given the availability of information on Class A level III hospitals (the most advanced hospitals within local provinces) in China, the health-care services index can be computed with a high level of accuracy. No ethical approval has been granted for the collection of these data, for it was exempt because the study extracted secondary data with no individual identifiers. And patient consent forms are not required for the study.

**Fig 1:**
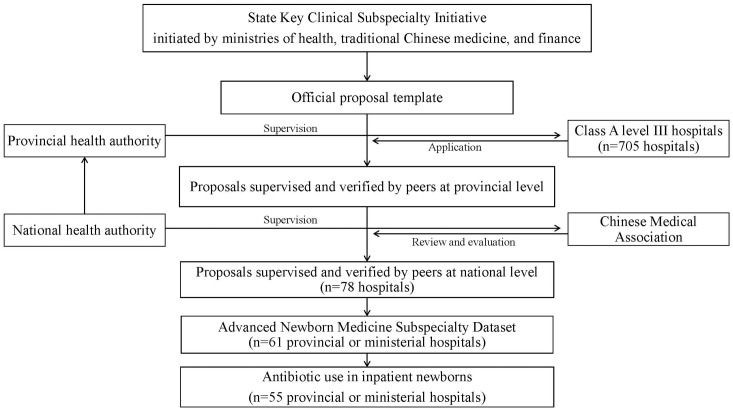
The procedure for data collection.

Of the 31 provincial districts in mainland China, three provinces (Shanxi, Hainan, and Tibet) that were relatively underdeveloped with respect to socioeconomic and health, did not submit applications[22]. In all, 61 newborn units from provincial or ministerial Class A level III hospitals affiliated with universities, were included in our preliminary dataset[20, 21]. Due to missing data from some hospitals, we selected 55 newborn units (hospitals) from 25 provincial districts (which account for 92% of the mainland population) and located in 28 cities (including one national capital, and 24 provincial capitals. See S1 Table). The procedure for data collection was shown in Fig 1. To ensure data quality, the authors had access to individual newborn unit information during and after data collection.

### Indicators and definitions

We selected indicators of health infrastructure, workforce, health service, and social and economic factors. Due to the unequal geographical distribution of the Chinese population and participating hospitals, we used the four broad geographical classifications according to the National Bureau of Statistics of China: Northern China (North China and Northeast China), East China, South Central China, and Western China (Southwest China and Northwest China). The newborn units were situated in three different hospital types: CH, GH, and MCH.

Data on health infrastructure and workforce reflect the situation at the end of 2010. Subspecialty beds are those registered at health authorities. The health workforce consists of full-time physicians and nurses with practicing certificates. Workforce indicators are number of physicians, number of nurses, nurse to bed ratio, physician to nurse ratio, proportion of physician with graduate degree, and proportion of nurses with at minimum a college certificate.

Given the fact that different newborn units may have different disease compositions, we chose pneumonia to preterm infant ratio, namely the proportion of pneumonia to preterm infants according to hospital discharge diagnosis codes, as a rough indicator of disease compositions. This approach was based on the facts that, within the investigation period, pneumonia and preterm infants were the two most common diseases in newborn units in China; and infants with pneumonia were prescribed antibiotic for treatment, while preterm infants with the gestational age between 34 and 36 weeks, who accounted for a majority of premature birth, may not be routinely prescribed antibiotic for treatment. Thus, a lower pneumonia to preterm infant ratio may suggest that a unit treated more immature babies, and a higher ratio may suggest that a unit was more likely to use antibiotics.

The primary outcome was the rate of inpatient antibiotic use, defined as the proportion of hospital discharges in which a patient received at least one dose of an antibiotic during the stay year[23, 24]. Annual inpatient discharge was the number of sick newborns treated by the physicians and nurses according to hospital inpatient management systems. Length of hospital stay was measured using the Organization for Economic Cooperation and Development (OECD) definition[25]. The data of urban per capita Engel coefficients were the situation in 2011, and from the China Statistical Yearbook[26].

### Analysis

We used means and standard deviations (SD) to explore the data. Arithmetic means of rates were used to compare antibiotic prescription rates across cities. We first conducted one-way analyses of variance (ANOVA) to compare different rates of antibiotic use across regions, hospital types, newborn ward capacity (grouped in tertiles: ≤50 beds, 51–100 beds, and >100 beds), as well as admission to physician or nurse ratio, proportion of physician with graduate degree, and ratio of nurse with college or above degree (each indicator divided in quartiles). We reduced the variables of workforce and workload to two components (nurse to bed ratio, and physician to nurse ratio) by using Factor Analysis with a principal components extraction. Due to the versatility of the generalized estimating equation (GEE) approach in analyzing longitudinal measures with repeated and categorical data[27], we conducted a GEE to estimate the parameters of a linear model for exploring the effects of different factors on antibiotic use rate over the three-year period, by selecting Hospital ID as a subject variable, period of year as a within-subject variable, newborn ward capacity as a factor variable. We adjusted for covariates: nurse to bed ratio, physician to nurse ratio, average hospital stay (day), pneumonia to preterm infant ratio, and urban per capita Engel coefficient. Data were analyzed using SPSS (version 23.0 Armonk, NY: IBM Corp.).

## Results

### General characteristics of the participating hospitals

Infrastructure and workforce of the participating hospitals were somewhat different (Table 1). Overall, GHs had the lowest levels of infrastructure and human resources, and the lowest number of inpatients discharged per unit (1664±964). CHs had the highest number of inpatients discharged per unit (3578±1592), and MCHs had the highest level of annual admissions per physician (156.4±58.4). Average hospital stay, and urban per capita Engel coefficient were also different when classified by geographic area.

**Table 1:** Characteristics of the participating hospitals.

	Total	Hospital types				Areas				
		GH (n = 25)	CH (n = 19)	MCH (n = 11)	P -value	Northern China^d^ (n = 13)	East China (n = 15)	South Central China (n = 17)	Western China^e^ (n = 10)	P -value
Number of subspecialty beds per unit^a^	84.4 (50.1)	57 (36.1)	109.8 (45.2)	102.9 (57)	<0.001	75.7 (45.3)	92.7 (52.3)	91.4 (57.4)	71.6 (41.3)	0.63
Number of physicians per unit^a^	22.7 (9.6)	18.0 (8.6)	27.8 (7.3)	24.5 (10.9)	0.002	21.7 (9.3)	24.2 (10.2)	24.2 (10.3)	19.2 (8.2)	0.54
Number of nurses per unit^a^	58.4 (30.6)	46.1 (28.5)	69.6 (30.3)	67.1 (27.5)	0.02	47.8 (24.8)	59.7 (34.7)	72.4 (33.3)	46.4 (16.4)	0.08
Nurse to bed ratio^a^	0.75 (0.30)	0.85 (0.38)	0.65 (0.15)	0.7 (0.18)	0.06	0.72 (0.34)	0.66 (0.16)	0.85 (0.26)	0.76 (0.42)	0.35
Physician to nurse ratio^a^	0.44 (0.19)	0.46 (0.19)	0.46 (0.22)	0.37 (0.10)	0.36	0.53 (0.26)	0.47 (0.21)	0.36 (0.08)	0.42 (0.12)	0.07
Proportion of physician with graduate degree^a^	0.71 (0.24)	0.83 (0.12)	0.63 (0.26)	0.57 (0.3)	0.002	0.73 (0.23)	0.70 (0.26)	0.76 (0.21)	0.61 (0.29)	0.46
Ratio of nurse with college or above degree ^a^	0.40 (0.22)	0.45 (0.21)	0.37 (0.24)	0.30 (0.17)	0.12	0.49 (0.30)	0.36 (0.21)	0.39 (0.19)	0.34 (0.13)	0.33
Average hospital stay (day)	9.6 (1.9)	10.0 (2.1)	9.5 (1.7)	9.1 (1.7)	0.09	9.3 (1.9)	9.8 (2.5)	10.1 (1.5)	9.0 (1.2)	0.04
Annual inpatients discharged per unit^b^	2639 (1505)	1664 (964)	3578 (1592)	3231 (1003)	<0.001	2207 (1077)	2750 (1596)	2936 (1583)	2527 (1778)	0.61
Annual admission to physician ratio^b^	130.3 (59.7)	107.6 (47.9)	145.1 (66.2)	156.4 (58.4)	0.03	126.1 (61.8)	123.8 (52.2)	130.9 (54.2)	144.6 (80.8)	0.85
Urban per capita Engel coefficient^c^	36.4 (2.8)	36.1 (2.9)	36.2 (3.0)	37.6 (2.0)	0.28	33.5 (2.3)	36.4 (2.4)	37.6 (2.1)	38.2 (2.1)	<0.001

Data are presented as mean (standard deviation). CH, child hospital; GH, general hospital; MCH, maternal and child hospital.

^a^ Data at the end of 2010.

^b^ Data in the 3 years of 2008–2011 period.

^c^ Data in 2011.

^d^ Including two geographical regions of North China and Northeast China according to the National Bureau of Statistics of China.

^e^ Including two geographical regions of Southwest China and Northwest China according to the National Bureau of Statistics of China.

### Comparisons of antibiotic use rates

Over the three years, the mean rate of antibiotic use was 65.7%±23.1% (median: 68.1%). The rate of antibiotic use varied from 13.7% to 100% across the 55 hospitals. There was a decline of 5.4% over the three-year span, but the difference was not significant: 2008 (67.8%±23.0%), 2009 (66.8%±23.0%), and 2010 (62.4%±24.2%).

The rates of antibiotic use differed significantly by hospital bed capacity (*p* = 0.023). Newborn units with more than 100 beds had the highest rate of antibiotic use (77.1%), compared to units with 50 or fewer beds (60.6%), and those with 51–100 beds (61.3%). We found no significant differences in antibiotic use across type of hospital, geographic area, physician or nurse academic degree, or annual admission to physician or nurse ratio (Table 2).

**Table 2:** Antibiotic use rates of inpatient newborns by classifications in 2008–2010.

Characteristics		Number of newborn medicine units	Percentage (%) of antibiotic-exposed newborns	P -value
Region	Northern China	13	58.6 (21.3)	0.45
	East China	15	71.1 (20.9)	
	South Central China	17	65.3 (24.7)	
	Western China	10	67.3 (25.8)	
Type of hospital	GH	25	62.3 (20.0)	0.94
	CH	19	71.5 (25.3)	
	MCH	11	63.2 (25.6)	
Newborn ward size	≤50 beds	17	60.6 (20.6)	0.02
	51–100 beds	22	61.3 (25.8)	
	>100 beds	16	77.1 (18.7)	
Admission to physician ratio	Quartile 1	14	68.4 (21.1)	0.12
	Quartile 2	14	57.9 (24.3)	
	Quartile 3	14	69.2 (19.5)	
	Quartile 4	13	67.3 (27.2)	
Admission to nurse ratio	Quartile 1	13	71.1 (19.9)	0.49
	Quartile 2	15	57.2 (20.8)	
	Quartile 3	13	68.1 (26.1)	
	Quartile 4	14	67.6 (24.7)	
Physicians with graduate degree	Quartile 1	14	73.2 (23.1)	0.78
	Quartile 2	13	60.6 (26.8)	
	Quartile 3	15	65.3 (22.1)	
	Quartile 4	13	63.1 (20.2)	
Nurses with an undergraduate or graduate degree	Quartile 1	14	66.2 (22.4)	0.14
	Quartile 2	14	62.4 (26.2)	
	Quartile 3	13	60.8 (23.1)	
	Quartile 4	14	73.0 (20.3)	

Data are presented as mean (SD). CH, child hospital; GH, general hospital; MCH, maternal and child hospital.

**Fig 2:**
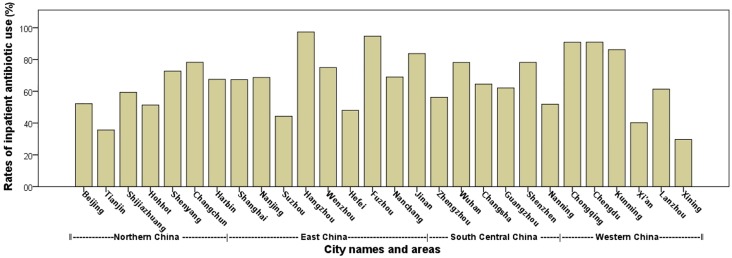
The rates of inpatient antibiotic use for newborns among cities. The bars illustrated the mean rates of inpatient antibiotic use among the participating hospitals from cities and areas in 2008–2010. Northern China included geographical regions of North China and Northeast China, and Western China included Southwest China and Northwest China.

There was a wide variation in rates of antibiotic use by city, as reflected by the city-related participating hospitals (Fig 2, and S1 Table), with the minimum in Xining (29.6%) and the maximum in Hangzhou (97.2%).

### Analysis of generalized estimating equation

Results of the generalized estimating equation showed that newborn ward capacity significantly predicts antibiotic use. Specifically, newborn wards with >100 beds, had a rate of antibiotic use that was 35.8% higher than that of newborn wards with ≤50 beds. Higher physician to nurse ratio, longer average hospital stay, and higher pneumonia to preterm infant ratio, also significantly and positively predicted antibiotic use (Table 3).

**Table 3:** Results of generalized estimating equation.

Parameter^a^	β	SE	95% Wald Confidence Interval	P-value
(Intercept)	-0.11	0.533	-10.153–0.934	0.84
Newborn ward: ≤50 beds	-0.358	0.103	-0.560 –-0.157	<0.001
51–100 beds	-0.278	0.086	-0.447 –-0.108	0.001
>100 beds	0^b^	—	—	—
Nurse to bed ratio	0.120	0.113	-0.102–0.342	0.29
Physician to nurse ratio	0.498	0.197	0.112–0.885	0.01
Average hospital stay (day)	0.002	0.001	0.001–0.002	<0.001
Urban per capita Engel coefficient	0.019	0.013	-0.007–0.045	0.15
Pneumonia to preterm infant ratio	0.003	0.001	0.001–0.004	<0.001

—, not applicable.

^a^Parameter: β weights for each index test derived from generalized estimating equation regression in which antibiotic use rate (0.01 = 1%) in inpatient newborns was entered in as outcome variable; SEs (standard errors) for β weights; Wald Confidence Interval for each β weight.

^b^Group of newborn ward with >100 beds as a reference to compare with other groups of newborn ward with ≤50 beds, and 51–100 beds.

## Discussion

Findings from our data on 55 advanced hospitals show that two-thirds (mean = 65.7%, SD = 23.1%) of inpatient newborns were prescribed antibiotic products during the period 2008 to 2010. A number of factors are shown to predict the rate of antibiotic prescription namely infrastructure (newborn ward capacity), workforce (physician to nurse ratio), and medical service uptake (pneumonia to preterm infant ratio, and average hospital stay).

The wide variation and overuse of antibiotic agents in newborns is a world-wide concern. One study of 127 NICUs in the USA found that overall antibiotic use varied 40-fold, from 2.4% to 97.1% of patient-days (median: 24.5%)[5]. Furthermore, a cohort of very low birth weight (VLBW) (401–1500g) neonates in the USA observed that 56% of 6215 VLBW neonates who survived at least 3 days received at least one course of antibiotic treatment, even if only 21% of the VLBW neonates had culture proven infection[28]. A high variability in dosage regimens of antibiotics was also reported in France[29]. A report from India also showed that up to 89% of neonates are prescribed antibiotics including newborns with no infection and unclear diagnoses[30]. Findings from our study suggest that antibiotic overuse in China is more serious than in the USA, but similar to India.

The study finding on contributing factors should be considered with care. It is understandable that the nature of diseases impacts antibiotic usage. For example, infants with pneumonia can be routinely treated with antibiotics in China, while premature births may not be an indicator of antibiotic use unless infection is suspected. The finding that antibiotic use was positively associated with high physician to nurse ratio, may suggest that shortage of nurses in newborn units increases the possibility of hospital infection, and thus antibiotic prescription for sick newborns. Meanwhile, studies prove that nurse to patient ratio influences many nurse-sensitive patient outcomes[31, 32], and nurse staffing at appropriate levels is exactly what the nature of newborn units calls for. On the contrary, shortage of nurses in China is very obvious regarding the number of nurses per unit (Table 1).

To our knowledge, this is the first report that newborn ward capacity influences antibiotic usage in inpatient newborns. This finding of the association between unit bed capacity and antibiotic usage can be supported by a national survey in China showing that hospitals with fewer than 300 beds had a significantly higher rate of antibiotic usage than those with more than 300 beds; and a systematic review of antibiotic utilization showing that hospital levels influenced the rate of antibiotic prescriptions among outpatients in China, where level I hospitals (primary care centers) were associated with higher antibiotic use than hospitals in high-level hospitals[6]. Furthermore, for preterm infants, a retrospective cohort study from 23 tertiary-level NICUs participating in the Canadian Neonatal Network during 2010–2012, also reported that a larger sized NICU was associated with higher risk of a composite outcome of either mortality or major morbidity[33]. In adult intensive care units, higher occupancy also can be associated with higher mortality and higher probability of severe adverse events[34, 35]. The potential impact of ward capacity on antibiotic use warrants further investigation, and may highlight the need for some agreement with careful plan, at a national or international level, about antimicrobial stewardship interventions and the most appropriate bed capacity in larger hospitals/units, to optimize antibiotic usage, as well as other medical outcomes.

There is much room left to reduce antibiotic overuse in newborn units. The report by The Centers for Disease Control and Prevention in the USA stated that one-third of antibiotic prescriptions in hospitals involved potential prescribing problems, such as unnecessary antimicrobial therapy, prescribing an antibiotic without proper testing or evaluation, or giving an antibiotic for too long [36, 37]. The National Action Plan for Combating Antibiotic-resistant Bacteria developed by a US governmental task force has set the goal to reduce inappropriate antibiotic use by 50% in outpatient settings and by 20% in inpatient settings by 2020[38]. The World Health Organization also has introduced a Global Action Plan on Antimicrobial Resistance[39]. Meanwhile, a recent prospective interrupted time-series study in the USA reported an overall decrease of 27% in antibiotic usage, yet there was no difference in safety outcomes between the intervention and baseline periods[40].

On a more encouraging note, our results show a decreasing trend in antibiotic usage over the three-year period, which constitutes a decrease of 17% compared to the results (83.4% in newborns) from 178 hospitals of China in 2001[8]. The efforts shared by health authorities and professionals in China included multiple strategies: 1) strengthening management in pharmaceuticals, sale and rational use of antibiotic agents; 2) involving experts and developing committees to integrate guidelines on antibiotic use in local context; 3) providing continuous education to hospital staff on infection control; 4) providing health education to general population; 5) monitoring bacterial resistance to guide clinical practice; and 6) supporting clinical studies on rational application of antibiotic agents[8]. Other measures included the implementation of an national essential medicines scheme in 2009 and zero-mark-up policy to terminate the economic incentives behind prescriptions; and the implementation of an antibiotic stewardship program in 2012 to ensure sustainable progress towards the rational use of antibiotic agents[41].

This study has several limitations. First, our results draw on administrative data on level III neonatal hospitals in China, meaning that the results do not reflect the situations of level one and level two hospitals. Second, we did not detail antibiotic prescribing patterns in the units to explore correlations with the presence of any indication, due to the fact that the available data were not acquired from an antibiotic-oriented site investigation. The strengths of the study are twofold. First, to our knowledge, it is the first study to systematically explore a range of factors that may contribute to the prescription of antibiotics. Second, the study examines usage over a three-year period, and thereby providing a baseline for future longitudinal studies.

## Conclusions

This retrospective analysis provides a comprehensive description of antibiotic use in inpatient newborns across advanced provincial and ministerial hospitals in China over a three-year period. Factors that contribute to antibiotic prescription include newborn ward bed capacity, physician to nurse ratio, disease composition, and average hospital stay. The data highlight the need to explore how subspecialty scale of newborn medicine potentially impacts the rate of antibiotic use among inpatient newborns.

## Supporting information

S1 Table List of participating hospitals. (DOCX)
