# Supplementary material for: Advanced neonatal medicine in China: Is newborn ward capacity associated with inpatient antibiotic usage?
Source: PLoS One. 2019 Aug 13;14(8):e0219630. doi: 10.1371/journal.pone.0219630 (PMC6692017; doi:10.1371/journal.pone.0219630)
Supplement: S1 Table — (DOCX) [file pone.0219630.s001.docx]

**SUPPLEMENTARY INFORMATION**

**S1 Table: List of participating hospitals**

| Hospital number | Regions | Provinces | Cities | Hospital types | Hospital number | Regions | Provinces | Cities | Hospital types |
| --- | --- | --- | --- | --- | --- | --- | --- | --- | --- |
| No01 | North China | Beijing | Beijing | CH | No29 | South Central China | Henan | Zhengzhou | CH |
| No02 | North China | Beijing | Beijing | CH | No30 | South Central China | Henan | Zhengzhou | MCH |
| No03 | North China | Beijing | Beijing | GH | No31 | South Central China | Hubei | Wuhan | GH |
| No04 | North China | Tianjin | Tianjin | MCH | No32 | South Central China | Hubei | Wuhan | MCH |
| No05 | North China | Tianjin | Tianjin | CH | No33 | South Central China | Hubei | Wuhan | CH |
| No06 | North China | Hebei | Shijiazhuang | CH | No34 | South Central China | Hubei | Wuhan | GH |
| No07 | North China | Hebei | Shijiazhuang | GH | No35 | South Central China | Hunan | Changsha | GH |
| No08 | North China | Inner Mongolia | Hohhot | GH | No36 | South Central China | Hunan | Changsha | CH |
| No09 | Northeast China | Liaoning | Shenyang | GH | No37 | South Central China | Hunan | Changsha | GH |
| No10 | Northeast China | Jilin | Changchun | GH | No38 | South Central China | Guangdong | Canton | MCH |
| No11 | Northeast China | Jilin | Changchun | CH | No39 | South Central China | Guangdong | Shenzhen | MCH |
| No12 | Northeast China | Heilongjiang | Harbin | CH | No40 | South Central China | Guangdong | Canton | MCH |
| No13 | Northeast China | Heilongjiang | Harbin | GH | No41 | South Central China | Guangdong | Canton | GH |
| No14 | East China | Shanghai | Shanghai | CH | No42 | South Central China | Guangdong | Canton | GH |
| No15 | East China | Shanghai | Shanghai | CH | No43 | South Central China | Guangdong | Canton | GH |
| No16 | East China | Shanghai | Shanghai | CH | No44 | South Central China | Guangxi | Nanning | MCH |
| No17 | East China | Jiangsu | Nanjing | CH | No45 | South Central China | Guangxi | Nanning | GH |
| No18 | East China | Jiangsu | Suzhou | CH | No46 | Southwest China | Chongqing | Chongqin | CH |
| No19 | East China | Zhejiang | Hangzhou | CH | No47 | Southwest China | Sichuan | Chengdu | MCH |
| No20 | East China | Zhejiang | Wenzhou | GH | No48 | Southwest China | Yunnan | Kunming | CH |
| No21 | East China | Anhui | Hefei | CH | No49 | Southwest China | Yunnan | Kunming | GH |
| No22 | East China | Anhui | Hefei | GH | No50 | Southwest China | Yunnan | Kunming | GH |
| No23 | East China | Fujian | Fuzhou | MCH | No51 | Northwest China | Shanxi | Xi'an | MCH |
| No24 | East China | Jiangxi | Nanchang | CH | No52 | Northwest China | Shanxi | Xi'an | GH |
| No25 | East China | Jiangxi | Nanchang | GH | No53 | Northwest China | Gansu | Lanzhou | GH |
| No26 | East China | Shandong | Jinan | GH | No54 | Northwest China | Gansu | Lanzhou | GH |
| No27 | East China | Shandong | Jinan | GH | No55 | Northwest China | Qinghai | Xining | MCH |
| No28 | East China | Shandong | Jinan | GH |  |  |  |  |  |

NOTE: Abbreviations: CH, child hospital; GH, general hospital; MCH, maternal and child hospital.
